# Chinese Medicines and Natural Medicine as Immunotherapeutic Agents for Gastric Cancer: Recent Advances

**DOI:** 10.1002/cnr2.2134

**Published:** 2024-09-05

**Authors:** Zhipeng Zhang, Ziqi Chen, Zujun Que, Zhihong Fang, Huirong Zhu, Jianhui Tian

**Affiliations:** ^1^ Institute of Oncology, Shanghai Municipal Hospital of Traditional Chinese Medicine Shanghai University of Traditional Chinese Medicine (TCM) Shanghai China; ^2^ Laboratory Center, Shanghai Municipal Hospital of Traditional Chinese Medicine Shanghai University of Traditional Chinese Medicine Shanghai China; ^3^ Shanghai Municipal Hospital of Traditional Chinese Medicine Shanghai University of Traditional Chinese Medicine Shanghai China; ^4^ Shanghai University of Traditional Chinese Medicine Shanghai China; ^5^ Clinical Oncology Center, Shanghai Municipal Hospital of TCM Shanghai University of TCM Shanghai China

**Keywords:** Chinese medicine, gastric cancer, immunotherapy, research progress

## Abstract

**Backgroud:**

According to the 2020 statistics from the World Health Organization’s International Agency for Research on Cancer (IARC), it is projected that there will be over 1 million new cases of gastric cancer (GC) patients worldwide in 2020, resulting in approximately 770 000 deaths. Gastric cancer ranks fifth in terms of incidence rate and forth in death rate among malignant tumors. Despite advancements in early diagnostic techniques, the incidence of GC has exhibited a marginal decline; nevertheless, the mortality rate remains elevated for advanced inoperable patients with no currently available efficacious treatment options.

**Recent Finding:**

Chinese medicine (CM) has emerged as an efficacious treatment for GC, gradually gaining acceptance and widespread usage in China. It exhibits distinctive advantages in the prevention and treatment of metastasis. CM and natural medicine possess the ability to elicit antitumor effects by augmenting immune cell population, enhancing immune cell activity, and improving the tumor immune microenvironment. CMs and natural remedies encompass a diverse range of types, characterized by multiple targets, pathways, and extensive pharmacological effects. Consequently, they have become a prominent research area among oncologists worldwide. Numerous studies have demonstrated that CM and natural medicine can directly or indirectly enhance innate immune system components (including macrophages, natural killer cells, and myeloid suppressor cells), adaptive immune system elements (such as T lymphocytes and regulatory T cells), relevant cytokines (e.g., IL‐2, IL‐4, IL‐10, TNF‐α), and PD‐1/PD‐L1 axis regulation, thereby bolstering the cytotoxicity of immune cells against tumor cells.

**Conclusions:**

This ultimately leads to an improved tumor immune microenvironment facilitating superior antitumor efficacy. This paper critically examines the role of CM and natural medicine in regulating immunotherapy for GC, aiming to establish a new theoretical framework for the clinical treatment and prevention of gastric cancer within the realm of CM.

**Abbreviations**
CM: Chinese medicine
CTL: cytotoxic T lymphocyte
EMT: epithelial‐mesenchymal transition
GC: gastric cancer
ICIs: immune checkpoint inhibitors
IL‐4: interleukin‐4
JPYZ: Jianpi Yangzheng decoction
mBYD: modified Bu‐zhong‐yi‐qi decoction
MDSCs: myeloid‐derived suppressor cells
MSCs: mesenchymal stem cells
NK: natural killer
OA: oleanolic acid
OS: overall survival
PFS: progression‐free survival
PSK: polysaccharopeptide
TAMs: tumor associated macrophages
Th1: helper T cells named type 1
Th2: helper T cells named type 2
TLR: toll‐like receptor
TME: tumor microenvironment
Tregs: regulatory T cells
TRM: *Trametes robiniophila* Murr

## 1 Introduction

Gastric cancer (GC) is a prevalent malignancy, with an estimated annual incidence exceeding 1 million cases, positioning it as the fifth most common neoplasm globally [1]. Due to the predominantly late‐stage diagnosis of gastric cancer patients, its high mortality rate has gradually propelled it to become the third leading cause of death. According to 2020 global statistics, an estimated 769 000 deaths were attributed to this disease [2]. Despite the advent of various chemotherapeutic agents, such as paclitaxel, oxaliplatin, and capecitabine which have demonstrated some extent of prolonging progression‐free survival (PFS) and overall survival (OS) [3], the natural course of the disease typically spans less than 1 year [4, 5] necessitating further enhancements in therapeutic effect. With the emergence of the immune era, immunotherapy has exhibited remarkable efficacy in treatment of various tumor types [6]. Despite the clinical benefit observed in numerous patients, treatment with immune checkpoint inhibitors (ICIs) has shown significant potential for therapeutic efficacy; however, a substantial proportion of patients exhibit resistance to such treatments or develop acquired resistance after an initial response, thereby impeding their potential benefits [7].

**FIGURE 1:**
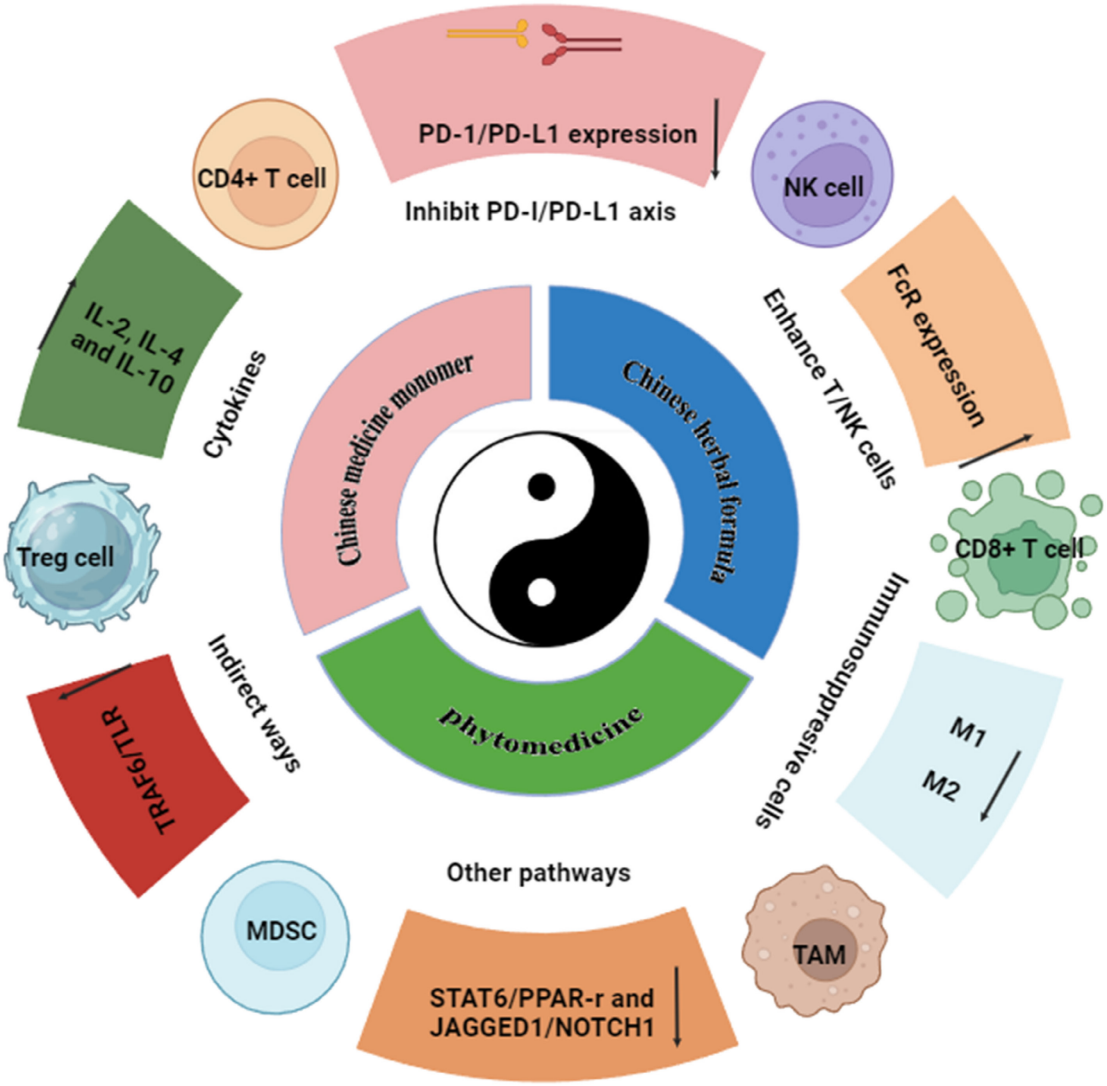
Chinese medicine regulates the immune microenvironment of gastric cancer [12, 14, 16, 20, 23].

The relative lag in immunological research and limited therapeutic options contribute to the underlying reasons for this phenomenon in gastric cancer, which currently only includes human epidermal growth factor receptor 2 (HER‐2) and programmed death ligand 1 (PD‐L1)‐targeted drugs. Clinical trials have consistently demonstrated that cytotoxic T‐lymphocyte‐associated protein 4 (CTLA‐4), vascular endothelial growth factor receptor (VEGFR), and other targeted drugs either alone or in combination with other agents exhibit restricted efficacy in patients with advanced gastric cancer compared with tumors such as lung cancer and colon cancer [8]. The primary cause of immunotherapy failure in gastric cancer is attributed to the induction of immune tolerance within the gastric cancer microenvironment. Consequently, addressing tumor‐induced immune tolerance becomes pivotal for achieving successful immunotherapeutic outcomes in gastric cancer. Recent research has highlighted the potential role of natural compounds, particularly phytocompounds, in preventing GC. Compounds, such as curcumin, resveratrol, and epigallocatechin gallate have significant anticancer properties against GC [9, 10]. Chinese medicine (CM) and natural medicine offer low toxic, safe, and efficacious therapeutic options with distinct advantages in modulating immune responses [11]. Currently, it has emerged as a prominent area of research focus. This review paper provides an overview of recent advancements in comprehending the mechanisms by which CM and natural medicine modulate the immune microenvironment in gastric cancer (Figure 1).

## 2 Progress of Research on Gastric Cancer Treatment

Data from multiple Phase III clinical studies, including Check Mate‐649, ORIENT‐16, RATIONALE‐305, KEYNOTE‐859, and GEMSTONE‐303, collectively provide robust evidence supporting the use of first‐line immunotherapy in advanced gastric cancer. These studies have demonstrated that the combination of PD‐1 inhibitors with standard first‐line chemotherapy significantly enhances OS in patients with advanced gastric cancer. This achievement has been acknowledged by international guidelines and incorporated into the standard first‐line treatment regimen for advanced gastric cancer. The CheckMate 649 trial was a phase III, multicenter, randomized, open‐label study that enrolled 1581 patients with previously untreated systemic, unresectable, HER‐2 negative, advanced gastric/esophagogastric junction cancer. The results of the study demonstrated a median OS of 13.1 months for the combination of nivolumab and chemotherapy compared with 11.1 months for chemotherapy alone. The combination of nivolumab with chemotherapy demonstrated a significant improvement in OS and PFS compared with chemotherapy alone, with a more pronounced effect observed in patients exhibiting PD‐L1 CPS ≥5. Based on these findings, the combination of nivolumab and chemotherapy has emerged as the world's first approved first‐line treatment for gastric cancer, marking a significant milestone in the era of immunotherapy for gastric cancer treatment [26]. The ORIENT‐16 trial was a randomized, double‐blind, Phase III clinical study, which enrolled 650 subjects with a median follow‐up of 33.9 months. The final analysis showed that sintilimab in combination with chemotherapy significantly reduced the risk of death in the CPS ≥5 population and in the overall population, with a 6.3‐month prolongation of median OS in patients with PD‐L1 CPS ≥5 and a 2.9‐month prolongation of median OS in the overall population. The 2‐ and 3‐year OS rates in the sintilimab‐combination chemotherapy group were 37.6% and 26.0%, respectively, in the overall population, and 43.6% and 29.0%, respectively, in the CPS ≥5 population; the overall safety profile of the treatment was good [27]. RATIONALE‐305 is a global, double‐blind, phase III study that compares the efficacy of tirilizumab in combination with an investigator‐selected chemotherapy regimen (TIS + ICC) versus placebo in combination with chemotherapy (P + ICC) for the first‐line treatment of GC/GJC. The results demonstrate that the addition of tirilizumab to chemotherapy significantly extends both median OS time (17.2 vs. 12.6 months) and median PFS time (7.2 vs. 5.9 months) in patients with PD‐L1 tumor area positivity score (TAP) ≥5%. Furthermore, this combination therapy improves patient objective response rate (ORR) (50.4% vs. 43.0%) and median duration of response (9.0 vs. 7.1 months). This protocol has been included in the 2023 Chinese Society of Clinical Oncology (CSCO) guidelines for gastric cancer [28]. The KEYNOTE‐859 trial was a randomized, double‐blind study that enrolled patients with HER2‐negative, locally advanced unresectable or metastatic adenocarcinoma of the stomach or gastro‐esophageal junction and known PD‐L1 status. The findings demonstrated that the addition of pembrolizumab to chemotherapy resulted in a modest increase in OS time, with an extension of only 1.5 and 1.6 months in the entire population and those with PD‐L1 CPS ≥1, respectively, while a more substantial improvement of nearly 4 months was observed in individuals with PD‐L1 CPS ≥10 [29]. The GEMSTONE‐303 trial is a randomized, double‐blind, multicentre Phase 3 clinical study designed to assess the efficacy of sugemalimab + CAPOX compared with placebo + CAPOX as first‐line treatment for patients with advanced G/GEJ adenocarcinoma. The findings demonstrate that the combination of sugemalimab and chemotherapy significantly enhances median PFS time (7.6 vs. 6.1 months), OS time (15.6 vs. 12.6 months), and ORR by 15.9% (68.6% vs. 52.7%) in patients with PD‐L1 expression ≥5%. Moreover, sugemalimab achieves a groundbreaking milestone as the world's first successful PD‐L1 inhibitor in gastric cancer and GEJC [30].

## 3 Chinese Medicine Regulates Immune Function to Elicit Antitumorigenic Effects

Over the course of 2000 years, CM has garnered widespread recognition as an epitome of medical practice in China and other Asian nations, throughout its evolution, CM has developed a distinctive and comprehensive framework encompassing unique theories, diagnostic approaches, and treatment systems. Distinguished by holistic principles and evidence‐based interventions, CM emphasizes the coordination and unity of the entire body to bolster individual resilience against diseases [31]. The comprehension of immune microenvironmental homeostasis and its role in tumorigenesis and development by contemporary medicine, as well as the etiology and pathogenesis of tumors and the evolution of their symptoms according to traditional Chinese medicine (TCM), are intricately intertwined [32]. Numerous medical practitioners propose a strong correlation between immunity and the body's positive energy, suggesting that an abundant presence of healthy qi in the body can function as a preventive measure against pathogenic factors. When healthy qi is deficient, the human body becomes susceptible to invasion by evil qi. Consequently, CM can exert control over tumor growth and metastasis by augmenting antitumor immunity. Particularly within the immunosuppressive tumor microenvironment (TME), CM also exhibits antitumor effects by enhancing immune response. In conclusion, the concept of CM treatment of gastric cancer aims to enhance the body's resistance to eliminate pathogenic factors, regulate the function of internal organs, and achieve a balance between yin and yang, as well as qi and blood in conjunction with immune modulation [33] and maintain the immune [34] balance intertwined together. Currently, CM scholars classify the treatment of gastric cancer into two aspects: tonification of the weakened aims to establish favorable conditions for dispelling the evil, while elimination of excess pathogens further safeguards positive qi. Both approaches are mutually complementary in clinical practice, with the aim of effectively treating the disease and saving patients.

## 4 CM and Natural Medicine Have a Regulatory Effect on Immune Cells

The regulation of the immune system by CM and natural medicine is characterized by a diverse and intricate interplay. Various CM and natural medicine compounds exert their effects on distinct subsets of immune cells, even within the same cell type. The naturally occurring compounds demonstrate a wide range of biological functionalities and induce diverse immunomodulatory responses, encompassing both innate and adaptive immunity [35]. Specifically, CMs have the potential to enhance antitumor effects by augmenting immune responses within the TME, including enhancing immune function and promoting immunocidal effects, as well as mitigating immunosuppression and preventing immune escape.

### 4.1 Chinese Medicine and Natural Medicine Modulate the Effects of CD4+ T and CD8+ T Cells

The peripheral T lymphocytes can be categorized into two primary subsets: CD4+ T cells and CD8+ T cells. These subsets, specifically the CD4+ T and CD8+ T cells, play a pivotal role in the context of antitumor immunity [36, 37]. CD4+ T cells secrete IL‐2 and IL‐15, thereby enhancing the activation of CD8+ T cell, sustaining CTL function, and additionally stimulating innate immune cells such as dendritic cells and natural killer (NK) cells [38]. Furthermore, certain CD4+ T cells subsets possess the capability to directly kill tumor cells [39]. CD4+ T cells can differentiate into type 1 helper T cells (Th1) and type 2 helper T cells (Th2). Th1 cells mainly secrete cytokines such as interferon‐γ, tumor necrosis factor‐α, and interleukin‐2, and play an important role in antitumor immunity by promoting the production of CTL. On the other hand, Th2 cells predominantly participate in humoral immunity through the secretion of cytokines like IL‐4, IL‐6, and IL‐13 to assist B lymphocytes in generating specific antibodies [40]. Dysregulation of Th1/Th2 signaling in gastric cancer cells leads to immune evasion [41]. CD8+ T cells release perforin and granzyme, which induce tumor cell death and lysis via the Fas/FasL pathway, ultimately resulting in apoptosis of target cells [42]. Therefore, the reduction of CD4+ and CD8+ T lymphocytes and the imbalance of Th1/Th2 play a key role in the occurrence and development of gastric cancer.

CM and natural medicine have the ability to enhance the activity of CD4+ T and CD8+ T lymphocytes and correct the Th1/Th2 imbalance. Wang et al. [12] found in an experiment on rats with gastric cancer, it was discovered that *Salvia divinorum* polysaccharides exhibited a significant capacity to enhance the production of anti‐inflammatory cytokines such as IL‐2, IL‐4, and IL‐10, suppress the secretion of pro‐inflammatory cytokines such as IL‐6 and TNF‐α, augment the cytotoxicity of CTL cells, and impede tumor cells proliferation. Li et al. [13] showed that polysaccharopeptide (PSK) has a regulatory role in both cellular and humoral immunity, with cellular immunity being the mainstay, which is shifted toward the Th1 type through activation of cytokines.

### 4.2 Chinese Medicine and Natural Medicine Enhance the Effect of NK

NK cells, renowned for their autonomous cytotoxicity against target cells, play a pivotal role as the principal effector cells in innate immunity's defense against cancer and exhibit remarkable heterogeneity within the microenvironment [43]. NK cells serve as the cornerstone of the host's immune defense against viral infections and malignant neoplasms [44]. NK cells can produce antigen‐independent immune responses against malignant cells. The killing effect of NK cells does not require activation by tumor‐specific antigens and is not restricted by histocompatibility complex I (major histocompatibility complex I, MHC I). Its killing pathways mainly include perforin, granzyme B pathway, and Fas/Fas L pathway, tumor necrosis factor‐α (TNF‐α) pathway, and antibody‐dependent cell mediated cytotoxicity (ADCC) [45]. In addition, NK cells can also secrete cytokines such as interferon‐γ (IFN‐γ), which play the role of regulatory cells of the immune system, activating T cells, macrophages, and so forth, to exert antitumor effects. The killing of tumor cells by NK cells depends on the interaction between the receptors expressed on their surface and the corresponding ligands on the surface of tumor cells. Furthermore, NK cells possess a natural cytotoxicity receptor (NCR) which binds to antigens located on the surface of tumor cells, resulting in their lysis [46]. Therefore, NK cells play a pivotal role in exerting control over the growth of gastric cancer and facilitating a potent antimetastatic effect.

According to literature reports, CM and natural medicine have been shown to augment the activity of NK cells. Lentinan, a bioactive compound derived from high‐quality Shiitake mushroom, exhibits remarkable efficacy in modulating the body's immune system. In gastric cancer patients, administration of lentinan effectively enhances both the quantity and functionality of NK cells, thereby improving their cytotoxicity against gastric cancer cells, and alleviating chemotherapy‐induced immunosuppression [14]. Previous studies have demonstrated that lentinan upregulates FcR expression, which may contribute to ADCC augmentation and increased NK cell‐mediated killing of tumor cells [15].

### 4.3 Chinese Medicine and Natural Medicine Can Inhibit MDSCs

Myeloid‐derived suppressor cells (MDSCs) were initially identified as “natural suppressor” cells in cancer patients three decades ago; however their association with tumors was not discovered until the 1990s [47]. Bronte et al. [48] animal studies have demonstrated the presence of CD11b and GR‐1 expression in mouse splenic tissues, with a mere 2%–4% of monocytes observed in normal mouse splenic tissues, while homozygous mice exhibit an elevated proportion exceeding 50%. Recent studies have revealed that murine bone marrow mesenchymal stem cells (MSCs) concurrently express molecules such as CD115, CD11b, IL‐4Rα, CD80, GR‐1, and CD32/16. Furthermore, these MSCs can be classified into granulocyte MDSCs (identified as cd11b + ly6g + ly6c low cells) and monocyte MDSCs (identified as cd11b + ly6g + ly6c high cells) based on variations in Ly6C and Ly6G expression [49, 50]. MDSCs play a crucial role in shaping the TME and exert potent immunosuppressive activity. Originating from the bone marrow, they migrate to the peripheral lymphoid organs and tumors of the tumor host, thereby fostering an immunosuppressive milieu conducive to tumor progression. Notably, MDSCs facilitate tumor cell survival, angiogenesis, and invasive dissemination of malignant cells into normal tissues [51, 52].

According to literature reports, TCM and natural medicine have been shown to possess inhibitory effects on MDSCs, thereby restoring the body's immune function. Curcumin, derived from the perennial herb *Curcuma longa* L., exhibits notable properties in cancer prevention and treatment [53, 54, 55, 56]. Tu et al. [16] revealed that curcumin effectively suppressed the activation and accumulation of MDSCs, disrupted the interaction between MDSCs and cancer cells, induced the differentiation of MDSCs, and impeded tumor proliferation. *Trametes robiniophila* Murr (TRM), an herbal medicine utilized in clinical settings to enhance immune response and enhance the efficacy of chemotherapy, has been reported by Xu et al. [17] to exhibit inhibitory effects on PMN‐MDSCs and PD1 + CD8+ T cells when combined with 5‐FU. Additionally, TRM was found to stimulate NK cells within the TME, and improve the cytotoxicity of 5‐FU against gastric cancer metastasis, ultimately leading to prolonged OS of patients. The role of exosomal PD‐L1 in regulating MDSC differentiation in GC has been reported, suggesting its crucial involvement. The CM compound (JPYZXZ) effectively remodels the immunosuppressive TME by inhibiting the exosomal PD‐L1‐mediated amplification of MDSCs, thereby mitigating GC progression [18].

### 4.4 Chinese Medicine and Natural Medicine Can Reverse TAM

Macrophages are a type of white blood cell derived from bone marrow stem cells [57]. They differentiate into mononuclear cells, and subsequently circulate through the bloodstream to various organs and tissues. Macrophages play a pivotal role in phagocytosis and digestion of pathogens and maintenance of tissue homeostasis [58]. Tumor‐associated macrophages (TAMs) infiltrating tumor tissues exhibit remarkable plasticity and heterogeneity. The polarization of TAMs toward the M2 type can be facilitated by colony‐stimulating factor 1 and interleukin‐4 (IL‐4), while toll‐like receptor (TLR) agonists, acting as proinflammatory cytokines, can induce the polarization of TAMs toward the M1 type [59]. Under the influence of IFN‐γ, IL‐6, and so forth, M1 macrophages can inhibit tumor cells by releasing soluble enzymes, TNF and IFN and activating T cell immune responses, showing proinflammatory, antigen presentation and antitumor effects [60]. However, M2 macrophages can be activated by IL‐10, IL‐13, and so forth. They can exert immunomodulatory and inhibitory effects and promote tumor progression through various mechanisms. Yuan et al. [61] found that M2 macrophages play a dominant role in TAMs and promote tumor growth, invasion, and metastasis. Recent studies have demonstrated that TAMs exhibit a propensity for phenotypic conversion toward the M2 phenotype, owing to their capacity to stimulate tumor cell proliferation, suppress the immune microenvironment within tumors, facilitate stromal remodeling, and expedite angiogenesis and lymphangiogenesis [62, 63]. Consequently, elevated levels of TAM are associated with poorer OS in several malignancies including gastric cancer [64].

Numerous studies have shown that CM and natural medicine can inhibit tumor growth and metastasis by regulating M1/M2 macrophages. Wu et al. [19] discovered that Jianpi Yangzheng decoction (JPYZ) effectively suppresses epithelial‐mesenchymal transition (EMT) transformation in gastric cancer by inducing a transition of macrophages from M2 to M1 phenotype. Zhao et al. [20] discovered that Dendrobium officinale polysaccharide induces the conversion of M2 macrophages into the M1 subtype through activation of the Notch1/Jagged1 and STAT6‐PPAR‐γ pathways, thereby effectively inhibiting gastric cancer growth. Jian‐pi‐yang‐zheng Decoction (JPYZ), a TCM used for advanced gastric cancer treatment, has demonstrated remarkable efficacy in patients. It has been reported that mJPYZ suppresses EMT in gastric cancer cells by modulating PI3Kγ‐dependent TAM reprogramming, ultimately leading to inhibition of gastric cancer progression and metastasis [21]. Furthermore, mJPYZ can attenuate the delivery of exosomal PKM2 from tumor cells to macrophages, mitigate the M2‐TAM differentiation induced by exosomal PKM2 in the TME, and ultimately impede the progression of gastric cancer [22].

### 4.5 Chinese Medicine and Natural Medicine Can Reverse the Reduction of Regulatory T Cells

Regulatory T cells (Treg) are immune negative regulatory cells that express CD4 and CD25. They play an important role in maintaining the body's immune homeostasis and also play a suppressive role in the TME [65]. Foxp3 is a Treg cell‐specific transcription factor that is highly expressed in Treg cells and is involved in the regulation of TCR signaling and transcription through interactions with other transcription factors [66, 67]. The expression of Foxp3 in gastric cancer tissues has been significantly correlated associated with tumor progression [68]. Throughout tumor development, chemokines secreted by tumor cells and macrophages recruit Tregs from the peripheral blood to the tumor site. Due to their potent immunosuppressive capabilities, Tregs enable tumor cells to evade immune surveillance [69]. Consequently, the occurrence, progression, and prognosis of gastric cancer are intricately linked to the presence of Tregs.

According to research, CM and natural medicine have been demonstrated to enhance the body's antitumor response by attenuating the release of immunosuppressive factors and reducing the abundance and functionality of Tregs. Xu et al. [23] demonstrated that modified Bu‐zhong‐yi‐qi decoction (mBYD) effectively regulates peripheral immunity and inhibiting the immune escape in tumors. The results demonstrated that CM effectively attenuates chemotherapy‐induced upregulation of CD8 + PD‐1+ T cells and Tregs in gastric cancer patients. Additionally, this formula directly enhances the proliferation, activation, and killing effects of T lymphocytes while prolonging survival in a gastric cancer xenograft mice model. Xu et al. [24] discovered through basic research that oleanolic acid (OA) can promote the balance of Treg/Th17 cells in GC by targeting IL‐6 with miR‐98‐5p.

## 5 Chinese Medicine and Natural Medicine Have a Synergistic Effect on Gastric Cancer Immunotherapy

CM, regarded as a national treasure, is distinguished by its low toxicity and minimal side effects, alongside its multitargeted approach that minimizes harm to the human body. The synergistic application of CM and natural medicine in conjunction with clinical medicine frequently yields unforeseen outcomes. Integrating CM and natural medicine with chemotherapy, radiotherapy, targeted drugs, and ICIs can enhance the efficacy of clinical treatments while mitigating their adverse effects and improve the patients' survival rates and quality of life [70]. The combination of CM and natural medicine has the potential to modulate the TME, regulate immune cell function, and enhance antitumor immunity by influencing the expression of the PD‐1/PD‐L1 signaling pathway. Therefore, TCM and natural medicine play a pivotal role in gastric cancer treatment, highlighting TCM's contribution toward promoting “positive energy”. Xu et al. [23] discovered in a basic study that mBYD inhibits the progression of gastric cancer by increasing the infiltration of tumor lymphocytes, decreasing the infiltration of PD‐1 and PD‐L1 in tumors, and decreasing the expression of PD‐1 in peripheral blood. Additionally, Xu et al. [17] found that the combination of TRM and 5‐FU significantly decreased the number of PMN‐MDSC and the expression of PD‐1 on CD8 + T cells and increased the number of NK cells in gastric cancer TME. Therefore, combination therapy can be an effective method of treating gastric cancer. Furthermore, Lu et al. [25] found from an in vitro study that OA could inhibit the IL‐1β/NF‐κB/TET3 axis activation leading to DNA demethylation which subsequently downregulated PD‐L1 expression (Table 1).

**TABLE 1:** Effects of CM and natural medicine on the immune microenvironment of gastric cancer.

Type of effect	Compounds/active ingredients	Modulation of immune modalities	Reference
CD4+ T/CD8+ T cells	A polysaccharide from *Salvia miltiorrhiza* Bunge	Promoted anti‐inflammatory cytokines (IL‐2, IL‐4, and IL‐10) production, inhibited proinflammatory cytokine (IL‐6 and TNF‐α) secretion	Wang et al. [12]
	Polysaccharopeptide	TRAF6/TLR immunosignal‐transduction pathways	Li et al. [13]
Natural killer cells	Lentinan	Increased FcR expression	Ina et al. [14]
	Lentinan	Increased FcR expression	Tani et al. [15]
Myeloid‐derived suppressor cells	Curcumin	Induces the polarization of MDSCs toward a M1‐likephenotype; suppresses the activation of Stat3 and NF‐κB in MDSCs	Tu et al. [16]
	*Trametes robiniophila* Murr	Regulating PMN‐MDSCs	Xu et al. [17]
	Jianpi Yangzheng Xiaozheng decoction	Inhibit MDSCs accumulation by reducing levels of exosomal PD‐L1	Wu et al. [18]
Tumor‐associated macrophages	Jianpi Yangzheng Xiaozheng decoction	Inhibiting the gastric cancer EMT transformation; inducing the phenotypic change in macrophages from M2 to M1	Wu et al. [19]
	Dendrobium officinale polysaccharide	The STAT6/PPAR‐r and JAGGED1/NOTCH1 signaling pathways	Zhao et al. [20]
	Modified Jian‐pi‐yang‐zheng decoction	Attenuated macrophage PI3Kγ and downstream NF‐κB signaling; inhibited gastric cancer cell EMT	Yuan et al. [21]
	Modified Jianpi Yangzheng decoction	Diminished the levels of PKM2 in exosomes derived from gastric cancer cells	Wu et al. [22]
Regulatory T cells	Modified Bu‐zhong‐yi‐qi decoction	Reduce regulatory T cells	Xu et al. [23]
	Oleanolic acid	Regulates the Treg/Th17 imbalance in gastric cancer by targeting IL‐6 with miR‐98‐5p	Xu et al. [24]
PD‐1/PD‐L1	Modified Bu‐zhong‐yi‐qi decoction	Reduction of PD‐1 and PD‐L1 expression	Xu et al. [23]
	*Trametes robiniophila* Murr	Decreased PD‐1 expression on CD8+ T cells	Xu et al. [17]
	Oleanolic acid	Blocked the IL‐1β/NF‐κB/TET3 axis	Lu [25]

## 6 Recent Research Progress in the Treatment of Gastric Cancer

The development of nanotechnology offers a novel avenue for the treatment of gastric cancer. Nanoparticles (NPs) have emerged as versatile and multimodal imaging agents and drug delivery carriers, making them promising candidates for innovative therapeutic strategies. Recent advancements in NPs and imaging techniques have revolutionized disease staging and response assessment in gastric cancer management. Currently employed diagnostic modalities encompass MRI, CT, single‐photon emission computerized tomography (SPECT), and positron emission tomography (PET). However, these imaging techniques suffer from limitations such as discordant imaging results, suboptimal pharmacokinetic profiles, rapid clearance rates, nonspecific distribution patterns, and potential adverse effects [71]. Hou et al. [72] developed a protease‐activated ratiometric fluorescent probe by utilizing fluorescence resonance energy transfer between a pH‐sensitive fluorescent dye and biocompatible Fe3O4 nanocrystals. This nanoprobe employed a peptide substrate of matrix metalloproteinase‐9 (MMP‐9) as a linker, connecting a particle bursting agent with a chromophore covalently attached to an antitumor antibody. In vitro cellular studies confirmed the activation of the nanoprobe by MMP‐9 secreted from gastric cancer cells, while the isolated chromophore effectively labeled tumor cells through covalent coupling with antibodies. Furthermore, in vivo imaging experiments combined with semiquantitative analyses demonstrated that this novel probe enabled sensitive pH mapping of the tumor microenvironment. Zhang et al. [73] used a specific target tyrosine kinase inhibitor for gastric cancer in the preparation of TNCA and recruited 484 patients with suspected gastric cancer to undergo CT examination as well as CECT using TNCA to diagnose gastric cancer, and evaluated the value of CECT using TNCA in the diagnosis of early gastric cancer, and the results showed that 302 cases of gastric cancer were detected by CECT using TNCA, which improved the sensitivity of diagnosis, suggesting that it can be used for early diagnosis of patients with suspected gastric cancer.

Advances in nanotechnology have facilitated the use of NPs in areas such as tumor therapy, imaging, diagnostics, and drug delivery [74, 75]. NP‐based drug delivery methods aim to improve conventional treatments for tumors as well as increase the efficiency of drug delivery [76]. NP delivery of drugs shows a number of advantages over traditional methods of chemotherapy drug delivery. Sharm et al. [77] developed a novel zinc oxide NP using aqueous extract of *Morus nigra* leaves as a capping and reducing agent for gastric cancer therapy. These NPs were prepared in the nanoscale size range and were effective in enhancing anticancer activity against gastric cancer cell lines (AGS) by decreasing mitochondrial membrane potential and increasing intracellular ROS levels, blocking the cell cycle and leading to apoptosis. In addition, silver NPs are one of the most widely used metallic NPs. Silver NPs synthesized by green synthesis methods from various natural sources such as Barbados aloe, Pagoda flower, and *Artemisia argyi* successfully inhibited the proliferation of gastric cancer cells (AGS and MNK45) and induced apoptosis, leading to effective treatment of gastric cancer [78].

## 7 Conclusion

The level of immunity in gastric cancer patients is intricately associated with tumor progression. The immune system exhibits autonomous stabilization, surveillance, and clearance functions, enabling the identification and elimination of foreign entities and senescent cells within the body [79]. The immune system has the ability to recognize specific antigens expressed on the surface of tumor cells and elicit an immune effect, thereby impeding tumor growth. However, relying solely on the body's immune function often falls short in completely eradicating tumor cells. In recent years, many studies have confirmed the existence of “immunoediting” between tumor cells and the body's immune system [80]. That is, tumor cells attenuate the body's antitumor immunity by altering the morphology of tumor antigens or reducing the number of tumor antigens. There are three stages in the process: the first stage is the “clearance” stage. The immune system kills and suppresses tumors faster than they grow, and the tumors are suppressed [81]. The second stage is the “balancing” stage. The immune system destroys tumor cells at roughly the same rate as the tumor grows, and the tumor is unable to progress further while the immune function is unable to clear the tumor cells [82]. The third stage represents the “escape” phase, during which tumor cells evade recognition by immune cells by altering the antigens on the cell surface. Immune escape from tumors is associated with immunosuppressive effects of immunosuppressive cells and tumors themselves in addition to downregulation of antigen expression on the tumor surface and alteration of antigen structure [83].

In recent years, there has been a global surge in the incidence and mortality of malignant tumors. Unlike traditional antitumor therapies such as surgery, radiotherapy, chemotherapy, and targeted therapy, immunotherapy has emerged as a new approach to combat cancer. Interestingly, CM adheres to the theoretical concepts of “Sufficient Healthy‐Qi inside the body will prevent invasion of pathogenic factors” and “strengthening the body resistance to eliminate pathogenic factors”, aligning with the current concept of immunotherapy for malignant tumors. CM and natural medicine play an indispensable role in the treatment of gastric cancer in many ways in China [84]. The intricate process involving multiple targets, cell types, and signaling pathways contributes to the formation and dynamics of TME. Whether they are active ingredients or compound formulas, all exert a certain regulatory effect on TME with diverse modes and points of action. Importantly, both CM and natural medicine not only impact tumor cells but also impacts immune cells, cytokines, and signaling pathways [85, 86]. The relationship and biochemical mechanism of TCM and natural medicine in regulating the TME necessitate further investigation, which is crucial for advancing our understanding. Despite the abundant medicinal compounds and potent biological activity found in TCM, there is currently a lack of approved clinical modulators for tumor immunotherapy. The intricate and diverse mechanisms underlying the immunomodulatory effects of these compounds and their active ingredients in gastric cancer include facilitating T‐cell proliferation, enhancing T‐cell and NK‐cell activity, regulating polarization of TAMs, as well as suppressing populations of regulatory T cells (Tregs) and MDSCs [87]. However, the targets and immunomodulatory activities of these active compounds remain elusive. Nevertheless, certain limitations persist in the current research: (1) Many studies have primarily focused on elucidating the molecular mechanisms through animal and in vitro experiments, while lacking clinical investigation reports. (2) The composition of CM soup exhibits instability during the decoction process, potentially leading to variations in clinical efficacy when employing CM compound prescriptions. (3) Numerous studies have demonstrated that various small molecules of TCM can regulate immune cells; however, it should be noted that immune cells are only a part of the TME and may not necessarily represent the central link in the antitumor effect of small molecules from TCM. (4) Most small molecule drugs are active ingredients derived from CM, with limited studies conducted on single medicines or compound prescriptions. Nonetheless, it is important to acknowledge that clinical applications of CM mainly involve compound prescriptions. (5) The mechanisms by which many CMs and their active ingredients regulate the body's immune system are still unclear. (6) Failure to fully exploit the advantages of CM and effectively harness its identification and treatment potential remains a challenge.

The author posits that future research on CM and natural medicine should prioritize strategies for enhancing drug utilization, improving drug targeting and accuracy, and optimizing immune cell activity stimulation. While CM and natural medicine hold significant potential in modulating crucial signaling pathways within the immune microenvironment of malignant tumors, their efficacy needs to be substantiated through multicenter, double‐blind, large‐sample clinical randomized controlled studies. Therefore, it is imperative to explore whether CM and natural medicine can achieve breakthroughs in gastric cancer immunotherapy or synergistically enhance clinical outcomes when combined with immunotherapy by further investigation. Moreover, immune cells play a pivotal role in the TME as well as the pathophysiological processes underlying tumor development. However, there is a paucity of clinical reports on CM and its small molecule drugs specifically targeting gastric cancer immune cells, thus highlighting the potential of utilizing CMs and their active ingredients as key directions for developing new clinical drugs within the field of TCM. Consequently, conducting extensive research on how CM and small molecule drugs exert modulatory effects in gastric cancer immunotherapy holds profound significance for expanding their clinical applications in treating patients with malignant tumors.

## Author Contributions

**Zhipeng Zhang:** writing – original draft, writing – review and editing. **Ziqi Chen:** writing – original draft, writing – review and editing. **Zujun Que:** visualization. **Zhihong Fang:** formal analysis. **Huirong Zhu:** writing – review and editing. **Jianhui Tian:** supervision, funding acquisition.

## Ethics Statement

The authors have nothing to report.

## Conflicts of Interest

The authors declare no conflicts of interest.

## Data Availability

The authors confirm that the data supporting the findings of this research are available within the manuscript.
